# Interacting partners of FEN1 and its role in the development of anticancer therapeutics

**DOI:** 10.18632/oncotarget.15176

**Published:** 2017-02-07

**Authors:** Chandrasekhar Kathera, Jing Zhang, Avilala Janardhan, Hongfang Sun, Wajid Ali, Xiaolong Zhou, Lingfeng He, Zhigang Guo

**Affiliations:** ^1^ Jiangsu Key Laboratory for Molecular and Medical Biotechnology, College of Life Sciences, Nanjing Normal University, Nanjing, China; ^2^ The Laboratory of Animal Genetics, Breeding, and Reproduction, College of Animal Science and Technology, Zhejiang Agriculture and Forestry University, Hangzhou, China

**Keywords:** protein-protein interaction, small molecular inhibitors, FEN1, posttranslational, modifications

## Abstract

Protein-protein interaction (PPI) plays a key role in cellular communication, Protein-protein interaction connected with each other with hubs and nods involved in signaling pathways. These interactions used to develop network based biomarkers for early diagnosis of cancer. FEN1(Flap endonuclease 1) is a central component in cellular metabolism, over expression and decrease of FEN1 levels may cause cancer, these regulation changes of Flap endonuclease 1reported in many cancer cells, to consider this data may needs to develop a network based biomarker. The current review focused on types of PPI, based on nature, detection methods and its role in cancer. Interacting partners of Flap endonuclease 1 role in DNA replication repair and development of anticancer therapeutics based on Protein-protein interaction data.

## INTRODUCTION

Irrepressible maturation of cells and their resistance to apoptosis are the important features of the cancer cells [1]. Various physiological and metabolic disturbances in the cells lead to the development of cancer [2]. Cancer is the leading cause of death in the world still we are in the confusion when it comes and understanding it. Each and every cancer has its own significant targets such as apoptotic signals, immune regulatory signals, proteins and enzymes, bodily functions like lungs, liver, bowel, etc. Approximately 14.1 million new cases of cancer in the world reported by GLOBOCAN in 2012, vast majority of deaths occurred by lung carcinoma or lung cancer in both men and women [3]. Many cancer cells developed resistance to chemotherapy, it kills drug-sensitive cells, but leaves behind a higher proportion of drug-resistant cells it leads tumor begins to grow again. So the academia and industrial research has focused on alternative treatment methods for cancer. Protein-Protein Interaction (PPI) is one of the major objectives in cancer research, widely used to investigate gene and protein related problems.

## PROTEIN-PROTEIN INTERACTIONS

**Figure 1:**
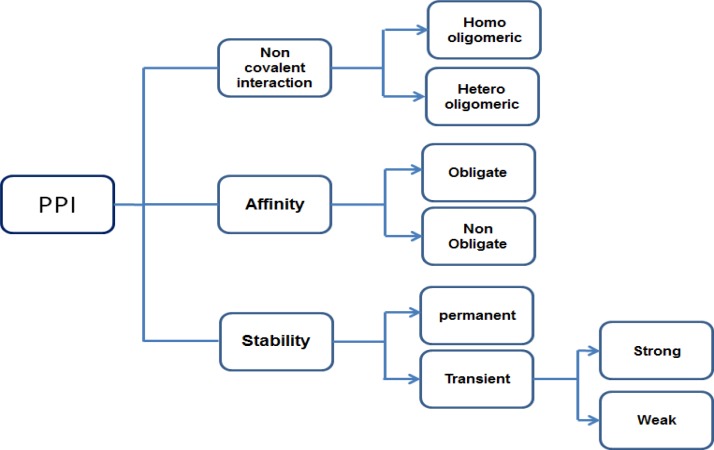
Different types of PPI based on nature

Protein-protein interaction (PPI) plays a crucial role in many biological actions such as intercellular, extracellular functions and apoptosis. Protein interactions help to understand the function performed by that protein, PPI should mediate key biological functions. Formations of the non-covalent bonds in between the protomers (smallest oligomeric protein) are the foundation for protein assembly, folding and Interaction. Physiological and environmental conditions influence the association and dissociation of the PPI complex in many biological processes [4]. PPIs can be classified based on the nature of interaction surface, stability and persistence. Based on interaction surface nature, PPIs are of two type's homo and hetero oligomeric interactions. These are macro molecular complexes, homo oligomers formed from only one type of protein subunits by non-covalent interactions, interaction between different protein subunits to form hetero-oligomers, hetero-oligomers are important to control several cellular functions. Based on PPI stability, they may be classified into obligate or non-obligate; affinity is the key point for differentiation between these two groups. Protein can form a stable complex without any other associated proteins in vivo, this type of complex proteins are called as non-obligate, some proteins can't form stable complex by its own, and such protein complexes are called obligate protein complexes. Based on persistence PPI may be transient and permanent. When compared to the nature of stability and life time, obligate and permanent interactions were easier to study than non-obligate and transient interactions. Transient interactions can be further divided into weak and strong (Figure 1). Based on the interaction surface of transient complex physicochemical and geometrical characteristics, transient interaction sites are selected as drug targets. These types of interaction sites may help to identify the evolutionary changes in small molecular inhibitors, which can serve as therapeutics for diseases.

**Figure 2:**
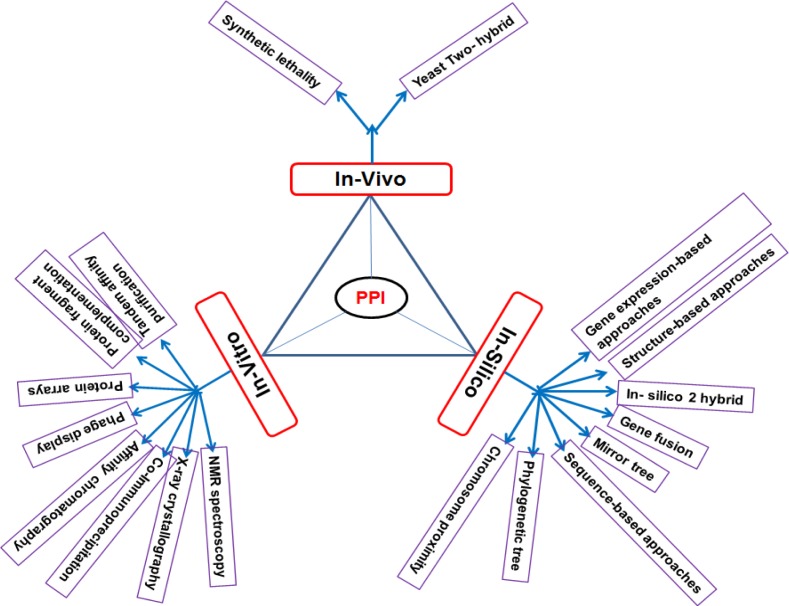
Different types of PPI network detection methods

Protein- protein interactions are act as a promising, challenging therapeutic development. Protein -protein interacts each other with its signaling nodes and network hubs, it transfers the signals along molecular network to attain an amalgamated biological output [5, 6]. By indicating details of potential binding partners they provide clear information of cellular function [7]. PPI network detection is required to understand the functions of unexplored proteins, PPI network detection methods can be classified into in-vitro, in-vivo and in-silico methods these methods can represented in the following (Figure 2).

## PROTEIN - PROTEIN INTERACTION ROLE IN CANCER?

Some Protein-Protein interaction acts as a heart of network highly connected to each other's, it provide valuable data to understand the cellular and metabolic function of a cell. The disfunction of some PPI may cause many diseases, including cancer [8]. Cancer related proteins interact with their partners through distinct interfaces it act as hubs [9]. These protein interactions showed high specificity and low affinity because of the shape, size, charge and hydrophobic nature of the protein [4, 10–11]. The protein interactions are absent in normal cells but present in cancer cells and also like absent in cancer cells but present in normal cells is termed as “gain and Loss of -function” [12]. These gain and loss of function influence the protein-protein interaction networks [13, 14].

Several protein- protein interactions imperative in neutralizing the tumor suppressor activity, like CDK4-pRB and MDM2-p53 and also allow binding some viral oncoproteins such as human papillomavirus E6, E7 which can induce the tumor development [15]. Some of the interactions like APC and FEN1 may promote the cancer, adenomatous polyposis coli (APC) and Flap Endonuclease both are tumor oppressors but when APC interact with FEN1, this interaction inactivate the tumor suppressor activity and promote the cancer through novel mechanisms [16]. Similarly APC interact with Pol-β, blocks its functions (Base Excision repair) Pol-β directed strand displacement synthesis and inhibits deoxyribose phosphate lyase activity in Long Patch (LP) and Short Patch(SP) Base Excision repair through different mechanisms [17–20]. Mutated FEN1 (FFAA FEN1) and PCNA complex may cause FEN1 deficiency, which influence on Base Excision repair and RNA primer removal, leading to numerous DNA breaks this develops aneuploidy- associated cancer [21].

P53 involved in governing of cellular processes, tumor suppression and inhibition of a variety of mechanisms like MDM2 (Mouse double minute 2 homolog) overexpression. MDM2 act as a primary negative endogenous regulator of p53. MDM2 and p53 regulate each other mutually through the auto-regulatory feedback loop via energizing of p53 transcribes MDM2, it leads to an increase MDM2 mRNA and protein and in turn, MDM2 protein binds to p53 on its N-termini and inhibits p53 mechanisms [22]. When MDM2 expression level altered in human cancer it involves different type of mechanisms like gene amplification, augmented translation [23, 24].

Recently identified a protein interaction between HER3 (human epidermal growth factor receptor 3) and DJ-1 (Protein deglycase DJ-1or Parkinson disease protein 7). HER3 is a tyrosine kinases receptor protein involved in signaling pathways for the control of multiple cellular processes like cell proliferation, organogenesis and tumorigenesis [25], HER3 has been reported in multiple cancers [26]. DJ-1 is a multifunctional protein involved in cell progression and proliferation, act as a redox regulated chaperone, cysteine protease and transcriptional co-activator, overexpression of DJ-1 has been reported in many cancers [27]. DJ-1 is a newly identified interacting partner of HER3 interacting with the cytoplasmic C-terminal tail of HER3. DJ-1 enhance the HER3 sensitivity of cancer cell against anti HER3 treatment. Overexpression of DJ-1 increased HER3 levels and promoted cancer cell proliferation in vitro and tumor growth in vivo [28].

## PROTEIN- PROTEIN INTERACTION IS A GATEWAY TO TREAT CANCER

Cancer cells become more resistant to drug treatment because of some mutations make these cells resistant to treatments such as chemo, biological and hormone therapies. Disadvantages of anti-cancer drugs that killed both cancer cells and healthy cells, traditional cancer treatment may cause Genotoxicity (chemical agents or radiation therapy that damages the genetic information within a cell causing mutations which may increase cancer) [29]. Now it's time to discover novel methods to treat cancer, like targeted therapies and network oncology, this can understate the risk of toxicity and reduce the cost of treatment [5]. Protein-protein interaction (PPI) is one of the best choice, it represents a vast class of therapeutic targets in both inside and outside the cell. It is a new target class that has been extremely challenging to convert to therapeutics, based on its properties, such as allosteric sites and hotspots, have been incorporated into drug design strategies [30–32].

Discovery of new biomarkers that leads to the development of clinical diagnosis, rapid development of advanced techniques supported new ways to identify network-based biomarkers PPI networks used as a biomarker for diagnosis of cancer. This is an important but ambitious task in biomedical field [33]. The most accepted biomarker discovery methods are depending on expression measurements of protein-protein associations [34]. Analyzing the protein expressions compared with controls and to identify network based biomarkers. Exploring these protein expressions profiling with some advanced tools designed to identify biomarkers [35].

Small molecule inhibitors block the protein-protein interaction it act as a new cancer therapeutic strategy. Targeting of PPI with small molecule inhibitor is a challenge, it provides a great potential for the discovery of chemical probes and therapeutic agents [36]. PPI networks provide a starting point for designing inhibitor compounds [37]. It plays a key role from intercellular communication to apoptosis of a cell life this represents the importance of PPI for targets of therapeutics [38]. Size, hydrophobic nature and adaptivity features of small molecules could actively adjust to bind a drug like molecule into the Protein-protein interaction [30].

## FEN1 AS A CENTRAL COMPONENT OF CELLULAR DNA METABOLISM

FEN1 a metallonuclease is composed of a nuclease domain and an extended C-terminal region (N, I and C terminal sections), which is responsible for important interactions with other proteins. FEN1 is known to rely on interactions with other DNA metabolic proteins for recruitment to different machineries for DNA replication, repair or degradation. Some of these association partners of FEN1 also stimulate its nuclease activities to facilitate efficient processing of various bifurcated DNA intermediates. To date, more than 34 proteins from various DNA metabolic pathways have been identified as interacting with FEN1. FEN1 is an essential enzyme, it possesses FEN, endo and exonuclease activities it involves in various DNA metabolic pathways like Okazaki fragment maturation, telomere maintenance, stalled replication fork rescue, long patch BER and apoptotic DNA fragmentation.

Okazaki fragments are short, newly synthesized DNA fragments that are formed on the lagging template strand during DNA replication. Maturation of Okazaki fragments is vital for DNA replication and cell proliferation. FEN1 can cleave these flaps by two ways i.e. Fen1 solely and FEN1-dna2 complex involved in this process, first stage FEN1 cleave the short flaps immediately, some of the flaps may escape from cleavage and thus become long, these flaps then bind RPA(replication protein A) which inhibits FEN1 cleavage and causes instability [39]. In the second way FEN1 bind with dna2 nucleases to process long flaps, dna2 can split the RPA binding flaps, then FEN1 can complete the cleavage process [40].

DNA is exposed to physical and chemical damaging agents which causes DNA damage, if it is not repaired, could leads to genetic mutations and subsequent genome instability and cancer [41,42]. BER (Base Excision Repair) is one of the major pathways in eukaryotic cells for processing DNA base damage caused by physical and chemical agents [43, 44]. FEN1 play a central role in LP-BER [45]. Although LP-BER functions in a PCNA- dependent and independent pathways utilizing plo-β, δ/ε. FEN1 stimulates the enzymatic activities of BER proteins to form a complex with (PCNA) [46].

**Figure 3:**
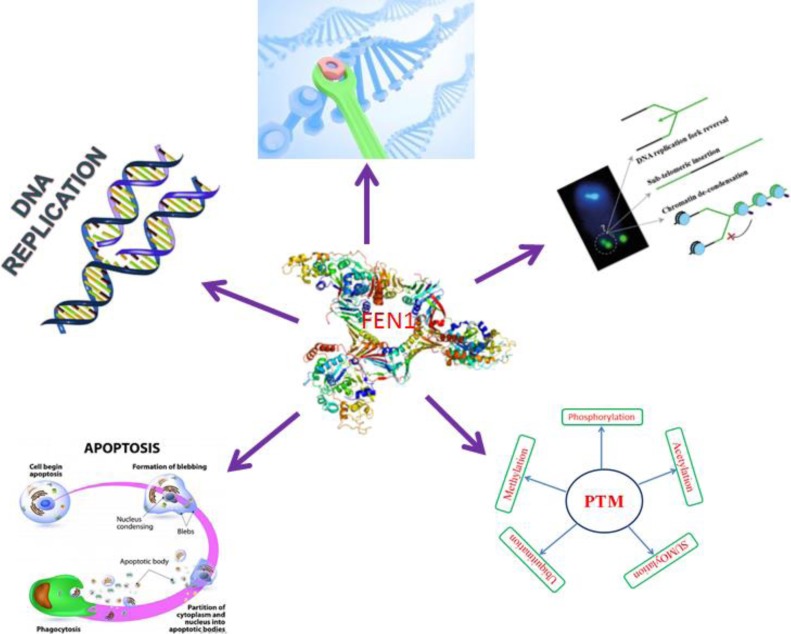
FEN1 central role in cellular DNA metabolism

FEN1 is vital for telomere stability, it plays an important role in the preservation of genomic stability and is maintained through the coordinated actions of telomere specific proteins, DNA repair and replication proteins [47,48]. FEN1 nuclease activity and the C-terminal interacting WRN protein are necessary for telomere stability [49, 50]. FEN1 is a target for post translational modifications; these modification changes can support the replication and repair process. FEN1 can be modified by methylation, acetylation, phosphorylation, ubiquitination and SUMOylation (Small Ubiquitin-like Modifier) [51–53]. Cellular DNA metabolism of FEN1 represented in (Figure 3).

## FEN1 PROTEIN -PROTEIN INTERACTION PARTNERS

FEN1 nucleases interact with many protein partners; reports suggest that the interacting partners may enhance the functions of FEN1. The extended C-terminus domain is important for protein-protein interaction, because it contains several arginine and lysine residues is critical for nuclease activity through involvement of substrate binding. To date, more than 34 proteins are known to interact with FEN-1, partners are interacted with FEN1 directly or indirectly to form a complex or multi protein complexes affect FEN-1 activities [54–56]. The interacting partners can be grouped into four different categories based on their function [21].

FEN1 and its interacting partners play a vital role in DNA replication, DNA synthesis is followed by polymerase α in eukaryotes, which synthesizes the initiator primer of RNA/DNA. RF-C (Replication Factor- C) also known as activator-1, is a five subunit protein complex, is required for DNA replication. RF-C loads the homotrimeric ring- shaped protein known as PCNA onto DNA. RNA/DNA primers are displaced to form a 5’ flap structure, which removed prior to ligating the remaining DNA segments [21, 57]. For Okazaki fragment maturation FEN1 can interact with PCNA, polymerase α /ε, DNA ligase, hnRNP A1 and RPA. In case of Yeast FEN1 interact with DNA2 sequentially cleave the RNA primers [58].

FEN1 also connected with WRN, BLM, RecQ, TRF2 and Chlr1, it implicated in DNA replication, recombination. FEN1, PCNA and WRN complex stimulates the GEN activity and cleaving the ssDNA region in the duplex DNA molecule and the complex is important for resolving stalled replication forks [59]. BLM protein is a member of the RecQ family of DNA helicases, involved in replication and also restrain the mutant DNA2 activity, its supports the FEN1 nuclease in Okazaki fragment maturation or adjust the activity to form a complex with FEN1 and DNA2 [60]. A novel interaction of the Bloom's syndrome (BLM) protein with FEN1 involved in Okazaki fragment processing, the complex stimulates both endo and exo nucleolytic cleavage activity of FEN1, BLM C-terminal domain that shares homology with the FEN-1 interaction domain of the Werner syndrome protein, a RecQ helicase family member homologous to BLM [61]. FEN1 interacted complex involved in telomere stability, chromosome segregation machinery and DNA replication, which include TRF2, WRN, TERT and Chlr1 [51, 62].

Environmental toxins, chemicals, drugs, stress, Inflammation and normal metabolic activities can cause the DNA damage resulting one million molecular lesions per day in a cell. Failure to resolve the damaged ends can lead to abnormal DNA replication and repair and is associated with genomic instability, mutagenesis and cancer [63]. In mammalian cells DNA repair process is of two types based on the length of the repaired flap, SN-BER or LP-BER. In BER, APE1 recognizes the apurinic/apyrimidinic residues; enzyme glycosylases remove a single nitrogenous base to create an AP site and generating a nick. Polymerase (β) enzymes then remove the damaged region using its exonuclease activity and synthesize a new strand [64]. APC interacts with and blocks FEN1 activity in Pol- β directed LP-BER[16]. Some FEN1 interacted proteins involved in both replication and repair which are PCNA, WRN. FEN1/WRN complex play a vital role to remove the 5'flaps in Long Patch Base Excision Repair (LP-BER) [62]. Complex of interact proteins Neil1, Pol-β, PCNA, APE1, Lig 1 and a complex of 9:1:1(Rad9-Rad1-Hus1) involved in LP-BER along with FEN1 [65,66]. Rad9-Rad1-Hus1 is a heteotrimeric protein like PCNA. In case of yeast non-homologous end-joining dsDNA break repair pathway find a new interacted proteins, Plo 4 and Dnl4/ Lif1 along with FEN1. FEN1 and interacted complex proteins involved in different DNA repair pathways indicates its importance in DNA repair.

FEN1 may involve in cell apoptosis, association of Endo G stimulates the mechanism of apoptotic DNA fragmentation, Endo G is a mitochondrial enzyme actively participate in caspase and DFF40 independent pathway [67]. Caspase and DFF 40 complex associated with some nuclear proteins such as H1 and HMG to promote cleavage of internucleosomal DNA Endo G. FEN1 with apoptotic proteins such as Endo G may be a key mechanism to switch FEN1's role from DNA replication and repair to apoptotic DNA fragmentation. FEN1 interaction with Endo G also greatly enhances its minor GEN and EXO activities, important for disposal of apoptotic DNA [68].

**Figure 4:**
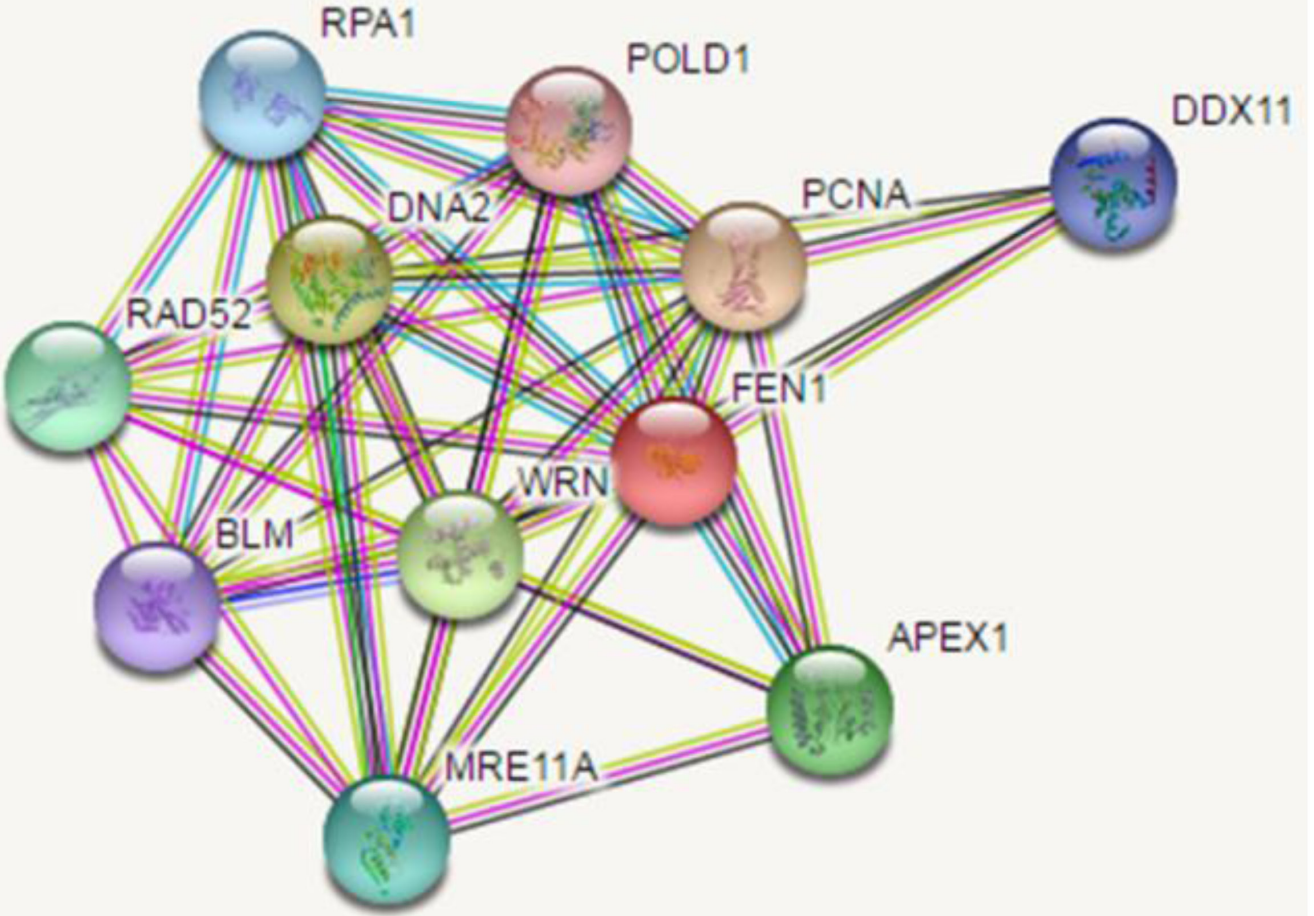
Interaction partners map of FEN1

FEN1 is a target for several posttranslational modifications, it can be modified by methylation, acetylation, phosphorylation, ubiquitination and SUMOylation [69, 70]. Many of the protein partners involved in FEN1 posttranslational modification they are P300, Cdk1-Cyclin A, Cdk2-Cyclin E and, UBE1/UBE 2M/ PRP19 [69,71]. FEN1 showed methylation at the R (arginine) 192 residue researchers confirmed that a link between FEN1 methylation and phosphorylation, methylation prevents FEN1phosporylation at Serine 187 residue. It influence on the PCNA binding during Okazaki maturation and replication process. Methylated FEN1 initially interact with PCNA and replaces pol δ to gain access to the flap. Following flap removal, FEN1 is phosphorylated which causes it to lose its interaction with PCNA and leave the substrate to provide access for DNA ligase-1. Acetylated FEN1 observed in Hela cells, p300 acetyl transferase interact with FEN1 both in-vivo and in-vitro, upon in-vitro condition four acetylated sites are identified and in-vivo condition three sites are identified, only one site is common in both in-vivo and in-vitro condition i.e. K375, the remaining are completely different [72]. In-vitro acetylated FEN1 reduced PCNA dependent nuclease activity and DNA binding affinity, but intact PCNA stimulating capacity [73]. FEN1 phosphorylated at Seine 187 in the late S phase by the cyclin A or Cdk2/ cyclin E complex, in-vitro phosphorylation FEN1 bound to its substrate with a similar affinity as the unmodified form, and its endonuclease activity is inhibited. Phosphorylation results in dissociation of FEN1 from PCNA. FEN1 shows multiple cell cycle -specific post-translational modifications [74], FEN1 levels is different in different stages of cell cycle when compared to the G2/M phases, S phase showed higher FEN1 level because of ubiquitination and SUMOylation. A complex interacts with FEN, UBE1/UBE2M/PRP19 complex ubiquinated FEN1 at K354 and SUMOylated at K168 [75]. All these interactions influence the FEN1 activities and its involvement in replication repair. FEN1 interacting partners are represented by the following (Figure 4) generated by STRING software.

## FEN1 ROLE IN THE DEVELOPMENT OF ANTICANCER THERAPEUTICS

Cancer cells turned as drug resistance, so anticancer drugs and small molecular inhibitor compounds development consider the key proteins involved in tumorigenesis and the mechanism [76]. FEN1 is a multifunctional structure specific endonuclease protein, it plays a key role in 5’ flap endonuclease (FEN), exonuclease (EXO), and gap-dependent endonuclease (GEN) activities. It plays an important role in maintaining genome stability, replication and repair [74]. Suppression of FEN1 leads to the retardation of DNA replication and accumulation of unrepaired DNA intermediates, resulting in DNA double strand breaks (DSBs) and apoptosis this may leads to cancer, DNA repair alters the risk of cancer [16]. Therefore, targeting FEN1 could serve as a potent strategy for cancer therapy [77]. Genomic DNA is constantly exposed to endogenous and exogenous insults, which cause genome instability if not properly repaired leads to mutations/cancer [78, 79]. Over-expression of FEN1 find in much type of cancer cells, over expression may involve tumor development it influence the cell proliferation and cell differentiation. In prostate cancer cells FEN1 act as a hormone refractory, cells are developed resistance against treatment, some of the small molecular inhibitors (SMI) inhibits the FEN1 activity and cells sensitive to the treatment [77, 80]. At the same time decrease the level of FEN1 in cells are more sensitive to treatment. This indicates FEN1 expression influence on tumor cell growth to anticancer drugs. Over expression of FEN1 observed in cancer cells like gastric, lung, neuroblastoma, pancreatic and prostate cancer and also observed the low level of FEN1 expression in some cancer cells like colorectal esophageal and gastric cancer. This information suggested that FEN1 is a potential biomarker for certain cancer types [81, 82]. The available data confirmed that the role FEN1 plays an important role in the development of small molecular inhibitors and anticancer therapeutic drugs.

## CONCLUSION AND FUTURE PROSPECTIVE

The present review focused on the role of PPI on development of therapeutics by considering the key proteins involved in tumorigenesis and mechanism. PPIs play a key role in cellular function and maintenance and also important to bind small molecular inhibitors. PPI play a significant role in identification of potential targets that can be considered to contribute toward next generation therapeutics [83]. PPI networks used as a biomarker for diagnosis of cancer, FEN1 is over expressed in many cancer cells, previously identified PPI data act as a biomarker to confirm the FEN1 levels in diagnosis sample when compare to normal, this information supports to early detection of diseases. FEN1 is a central component in cellular mechanism; hopefully it plays a lead role in drug design and treatment. Cancer cells turned as drug resistance, so now focus on new manner to develop small molecular inhibitors and anticancer drugs based on the PPI data. It's an alternative and advanced way to develop new therapeutics.

## Abbreviations

FEN1: Flap Endo nuclease-1
PPI: Protein-Protein Interaction
PTM: Posttranslational Modifications
MDM2: Mouse double minute 2 homolog
BER: Base Excision repair
Pol-β: polymerase beta
